# Microarray analysis identifies candidate genes for key roles in coral development

**DOI:** 10.1186/1471-2164-9-540

**Published:** 2008-11-14

**Authors:** Lauretta C Grasso, John Maindonald, Stephen Rudd, David C Hayward, Robert Saint, David J Miller, Eldon E Ball

**Affiliations:** 1 Centre for the Molecular Genetics of Development, Research School of Biological Sciences, Australian National University, Canberra, Australia; 2 Centre for Mathematics and its Applications, Mathematical Sciences Institute, Building 27, Australian National University, Australia; 3 Turku Centre for Biotechnology, Tykisokatu 6, 20521 Turku, Finland; 4 Centre for Molecular Genetics of Development & ARC Centre of Excellence for Coral Reef Studies, James Cook University, Townsville, Australia

## Abstract

**Background:**

Anthozoan cnidarians are amongst the simplest animals at the tissue level of organization, but are surprisingly complex and vertebrate-like in terms of gene repertoire. As major components of tropical reef ecosystems, the stony corals are anthozoans of particular ecological significance. To better understand the molecular bases of both cnidarian development in general and coral-specific processes such as skeletogenesis and symbiont acquisition, microarray analysis was carried out through the period of early development – when skeletogenesis is initiated, and symbionts are first acquired.

**Results:**

Of 5081 unique peptide coding genes, 1084 were differentially expressed (P ≤ 0.05) in comparisons between four different stages of coral development, spanning key developmental transitions. Genes of likely relevance to the processes of settlement, metamorphosis, calcification and interaction with symbionts were characterised further and their spatial expression patterns investigated using whole-mount in situ hybridization.

**Conclusion:**

This study is the first large-scale investigation of developmental gene expression for any cnidarian, and has provided candidate genes for key roles in many aspects of coral biology, including calcification, metamorphosis and symbiont uptake. One surprising finding is that some of these genes have clear counterparts in higher animals but are not present in the closely-related sea anemone *Nematostella*. Secondly, coral-specific processes (i.e. traits which distinguish corals from their close relatives) may be analogous to similar processes in distantly related organisms. This first large-scale application of microarray analysis demonstrates the potential of this approach for investigating many aspects of coral biology, including the effects of stress and disease.

## Background

Cnidarians are the simplest animals at the tissue level of organization, and are of particular importance in terms of understanding the evolution of metazoan genomes and developmental mechanisms. Members of the basal cnidarian Class Anthozoa, which includes the sea anemone *Nematostella *and the coral *Acropora*, have proved to be surprisingly complex and vertebrate-like in terms of gene repertoire [1-3], and are therefore of particular interest. Scleractinian corals are also of fundamental ecological significance in tropical and sub-tropical shallow marine environments as the most important components of coral reefs. Surprisingly, both the general molecular principles of cnidarian development and many aspects of the functional biology of corals are only poorly understood. Whole genome sequences are now available for both the textbook cnidarian *Hydra magnipapillata *and the sea anemone *Nematostella vectensis*. However, corals are distinguished from *Nematostella *and other cnidarians by the presence of an extensive skeleton composed of calcium carbonate in the form of aragonite. The ability to carry out calcification on a reef-building scale is enabled by the obligate symbiosis between scleractinians and photosynthetic dinoflagellates in the genus *Symbiodinium*.

Expressed Sequence Tag (EST) projects carried out on *Acropora millepora *and *Nematostella vectensis *have provided insights into the evolution of animal genomes [2,3]. The latter publication, based on ca 5800 unigenes from the coral *Acropora *and 10,500 unigenes from the sea anemone *Nematostella*, revealed the surprisingly rich genetic repertoire of these morphologically simple animals. The genomes of anthozoan cnidarians encode not only homologs of numerous genes known from higher animals (including many that had been assumed to be 'vertebrate-specific'), but also a significant number of genes not known from any other animals ('non-metazoan' genes; [3]). This picture of genetic complexity has been augmented by the recently completed whole genome sequence (WGS) of *Nematostella vectensis *[1], for which approximately 165,000 ESTs are now available. Similar resources exist for *Hydra magnipapillata *[4,5] although the much larger genome size of this organism has consequences for the completeness of the assembly. Both of these other cnidarians not only lack a calcified skeleton, but also do not enter symbioses. Entry into a symbiosis can have profound effects on gene expression patterns, with changes to immune function, and to many metabolic functions including CO_2_ cycling, nutrient cycling, metabolite transfer and reactive oxygen quenching [6,7]. The phylogenetic position of *Nematostella *makes this a particularly useful comparator because both *Nematostella *and *Acropora *are classified into the anthozoan subclass Hexacorallia (Zoantharia).

**Figure 1:**
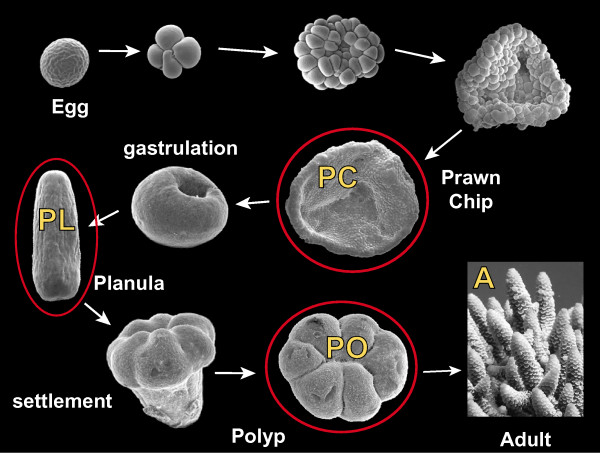
**Scanning electron micrographs of developmental stages in the *Acropora millepora *lifecycle**. At spawning egg-sperm bundles are released by the colony and float to the surface, where they break up into individual eggs and sperm. Upon release and fertilization of the egg, cell division first produces a spherical bundle of cells which then flattens to form a cellular bilayer called the prawnchip (PC). Following gastrulation the spherical gastrula elongates to a pear shape as cilia develop. Further elongation produces a motile presettlement planula larvae (PL), possessing a highly differentiated endo- and ectoderm and an oral pore. Upon receipt of an appropriate cue, the larva settles and metamorphoses, forming the primary polyp (PO). Following calcification, symbiont uptake, and growth and branching, the adult colony is formed (A). The stages labelled with yellow letters represent those from which RNA was extracted, labelled and hybridized to the slides. Stages circled in red are those from which ESTs were spotted onto the slides.

Information and resources relevant to microarray studies on corals have recently been summarised [8]. Few precedents exist for the approach used here; the most directly relevant previous study is an array experiment comparing symbiotic and aposymbiotic sea anemones [9]. To gain insights into the molecular bases of coral development, including nematocyst formation, metamorphosis, and the processes of symbiont uptake and calcification, developmental microarray experiments were carried out using 12000 spot cDNA arrays representing 5081 *Acropora millepora *unigenes which, based on the EST sequence, are predicted to give rise to a bona fide protein. Four stages of coral development were compared, spanning the major transitions of gastrulation and metamorphosis (Figure 1). These comparisons, which constitute the most comprehensive analysis of the development of any cnidarian to date, provide insights into the overall dynamics of the transcriptome during development as well as candidate genes for roles in metamorphosis, calcification and symbiont uptake. Spatial expression patterns were determined for many of the candidate genes identified in the array experiments. Comparisons with *Nematostella*, *Hydra *and other animals imply that nominally coral specific processes are executed by both conserved and novel (taxon-specific) genes, and suggest some intriguing parallels with other systems.

## Results

### The identification and composition of synexpression clusters

Of the 5081 unigenes giving rise to predicted peptides that are represented on the arrays, a total of 1084 unigenes (2462 spots) were found to be up- or down-regulated (P = < 0.05) between any two consecutive stages. The microarray results were validated by virtual northern blots. The results for eight arbitrarily chosen clones are shown in Additional File 1; in each case the observed expression pattern corresponds with the microarray results.

**Figure 2:**
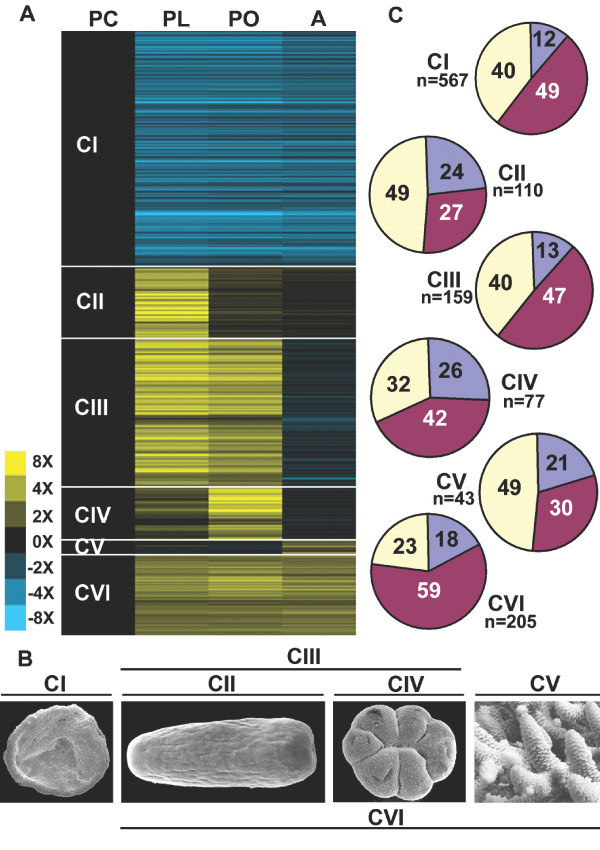
**Summary of microarray results**. (A) Graphical representation of the six expression clusters: yellow corresponds to upregulation and blue to downregulation. Each row corresponds to an EST and each column to a developmental stage as labelled in Figure 1. Clusters I-VI consist of genes with their highest expression in the prawnchip, presettlement, presettlement and post-settlement, post-settlement, adult, and post-gastrulation stages, as diagrammed in (B). Presettlement orientation is oral to the left, postsettlement orientation is oral pointing out of the plane of the page. (C) Pie charts classifying the genes in each cluster into unique genes (blue-unique to *Acropora*), core genes (purple-matching a database entry in non-Metazoa, Radiata and Bilateria) and other (light yellow- any combination of any two of non-Metazoa, Radiata or Bilateria). Note that whilst 1084 unigenes were differentially expressed, the total number of unigenes in clusters is 1161. This is because 70 unigenes fall into two or more clusters, possibly due to the existence of splice variants for some unigenes.

Cluster analysis identified six major synexpression clusters (Figure 2A) which map onto the major stages of coral development (Figure 2B). Three of these clusters (CII, CIII and CIV) are of most interest from the perspective of coral-specific biology. Candidates for roles in nematocyst development, receipt of settlement cues and the implementation of metamorphosis may be represented in cluster II (genes up-regulated in planula) or cluster III (genes up-regulated in planula and primary polyp). Similarly, genes involved in the early stages of calcification are predicted to occur in cluster IV (genes up-regulated in primary polyp) and cluster III (genes up-regulated in planula and primary polyp). These same two clusters (CIII and CIV) may also provide candidates for roles in the establishment of symbiosis. Two other synexpression clusters (CI and CVI) are of more general developmental interest. The largest, cluster I (genes down-regulated after embryogenesis), consists of 567 unigenes whose transcript levels decreased after gastrulation and remained low (Figure 2A). Cluster V (genes up-regulated in adult) consists of only 43 unigenes. The small size of this cluster may be due to the absence of adult material amongst the cDNAs spotted on the array and therefore presumably reflects only a small proportion of the total number of genes that are up-regulated in adult coral.

Functional breakdown data for the genes in these clusters are summarised in Table 1. Overall, approximately 15% of the differentially expressed genes are coral specific (no match to database sequences at < 1 × e^-5^), but the relative proportion of these nominally taxon-specific genes varies widely between the synexpression clusters. Clusters II (genes up-regulated in planula) and IV (genes up-regulated in primary polyp) contained the highest proportions (23.5% and 26%; 26 and 20 unigenes respectively) of unique genes, but these accounted for only 12% of cluster I (Figure 2C). Conversely, cluster VI contained the highest proportion (59%) of 'core' genes, which are defined as genes represented in animals and other kingdoms (Figure 2C). The proportion of *Acropora *unigenes matching only to other cnidarians was relatively constant across clusters, cluster VI (7%; 14 unigenes) being somewhat below the 9–11.5% range of the other clusters (data not shown).

**Table 1:** Percentage of unigenes within expression clusters in each functional category.

**Category**	**Category Description**	**Prawn-chip (567)**	**Planula (110)**	**Planpol (157)**	**Polyp (77)**	**Adult (43)**	**House-keeping (205)**
***A***	***Functions that many kinds of cells use***						
AI	Transportation and binding proteins for ions and other small molecules	1.4	0.0	4.3	6.4	4.7	3.4
AII	RNA processing, polymerising, splicing, and binding proteins and enzymes	7.0	1.8	2.5	1.3	4.7	0.5
AIII	Cell replication; histones, cyclins and allied kinases DNA polymerases, topoisomerases, DNA modification	9.8	0.0	0.6	0.0	0.0	0.0
AIV	Cytoskeleton and membrane proteins	3.9	4.5	6.2	3.8	4.7	6.8
AV	Protein synthesis cofactors, tRNA synthetase, ribosomal proteins	2.5	7.2	3.1	0.0	2.3	29.1
AVI	Intermediary synthesis and catabolism enzymes	8.3	13.5	27.2	17.9	11.6	16.0
AVII	Stress response, detoxification and cell defence proteins (GFPs in here)	0.4	2.7	1.2	1.3	0.0	1.9
AVIII	Protein degradation and processing, proteases, apoptosis related	4.0	3.6	2.5	2.6	0.0	1.5
AIX	Transportation and binding proteins for proteins and other macromolecules	3.3	1.8	0.6	5.1	0.0	1.5
***B***	***Cell-cell communication***						
BI	Signalling receptors, including cytokine and hormone receptors and signalling ligands	1.9	3.6	4.9	2.6	2.3	0.5
BII	Intracellular signal transduction pathway molecules including kinases and signal intermediates	10.5	0.9	5.6	1.3	9.3	0.5
BIII	Extracellular matrix proteins and cell adhesion	0.9	14.4	6.8	7.7	4.7	3.4
**C**	***Transcription factors and other gene regulatory proteins***						
CI	Sequence-specific DNA binding proteins	3.3	0.0	0.6	1.3	4.7	1.0
CII	Non-DNA-binding proteins with positive or negative regulatory roles	1.2	0.0	0.0	0.0	0.0	0.0
CIII	Chromatin proteins other than AIII with regulatory function	0.7	0.0	0.6	0.0	4.7	0.0
D	***Not enough information to classify***	40.8	45.9	33.3	48.7	46.5	34.0

The number of unigenes in each cluster is in brackets.

Approximately 10% of cluster I (genes down-regulated after embryogenesis) consists of genes in functional category AIII, genes involved in cell replication [10], probably reflecting the extent to which cell proliferation dominates early embryogenesis. 29.1% of cluster VI (genes up-regulated after embryogenesis) were classified into functional category AV: protein synthesis cofactors, tRNA synthetase, and ribosomal proteins, whereas all other clusters contained very few genes in this category. 27.2% of cluster III (genes up-regulated in planula and primary polyp) were classified into AVI: Intermediary synthesis and catabolism enzymes; this is significantly more than in any other cluster.

Planula larvae are primarily dependent upon stored lipid, whereas the energy requirements of adult corals are often largely met by photosynthetic products exported from their dinoflagellate symbionts. These physiological changes are reflected by shifts in the coral transcriptome. For example, lipases are highly represented amongst the planula ESTs, but strongly down-regulated thereafter. Also of note are dramatic differences in representation of genes in category BII (intracellular signalling) between cluster I (10.5%) and cluster II (0.9%), and in genes in category BIII (extracellular matrix and cell adhesion) between cluster I (0.9%) and cluster II (14.4%). These shifts, and the sharp spike in expression of ECM and cell adhesion genes, are associated with the transition from an undifferentiated proliferative stage and the emergence of differentiated cell types.

### Lectins related to sea cucumber CEL-III are strongly expressed during metamorphosis in *Acropora*

Whilst our understanding of metamorphoses in marine invertebrates is very incomplete, in several cases key molecules implicated in the underlying processes have been identified, and these include lectins [11,12]. Studies of coral settlement and metamorphosis have indicated that the inductive morphogenetic cue is exogenous/environmental and, whilst the exact structure of the metamorphosis inducing morphogen remains elusive, lipopolysaccharides are prime candidates [13] suggesting that cell surface recognition by coral larvae may be mediated by lectins. Lectins are therefore of particular interest as candidates for roles in settlement and metamorphosis as well as in other developmental processes including the uptake of *Symbiodinium *(see below). Indeed, a mannose-binding lectin has recently been described from *A. millepora *which binds both bacteria and *Symbiodinium *and may therefore have roles in both immunity and symbiosis [14]

**Figure 3:**
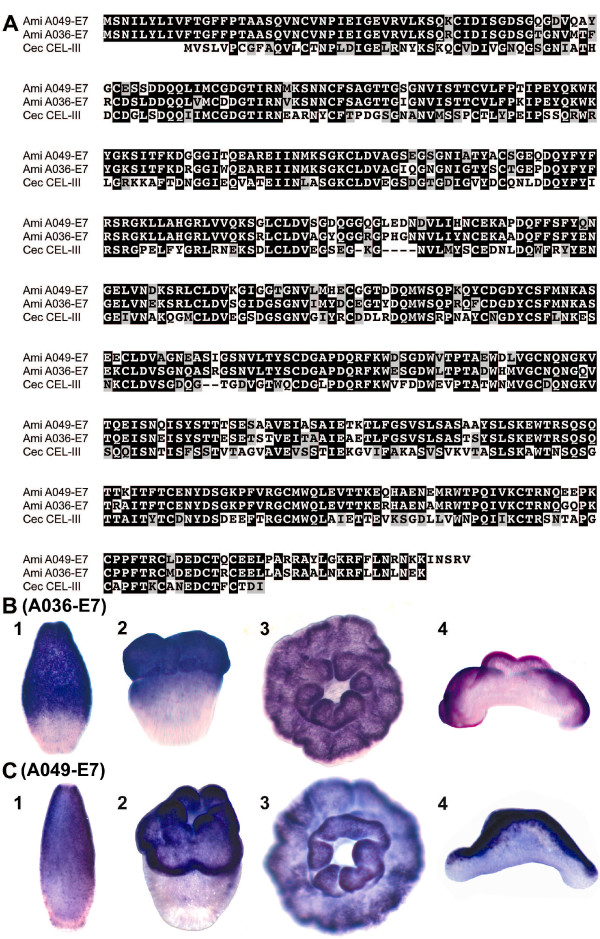
**Sequence comparison and whole mount in situ hybridization of lectin coding genes A036-E7 and A049-E7**. (A). Alignment of A036-E7 and A049-E7 amino acid sequences with *C. echinata *CEL-III reveal that they are 82.1% identical (90.6% similar) to one another and 50.4% (65.1%) and 48% (64%) to CEL-III respectively. Black boxes represent identities and grey shaded boxes similarities. Localisation of A036-E7 (B) and A049-E7 (C) transcripts (dark purple) in presettlement planula larvae (1), metamorphosing larvae (2), and postsettlement polyps viewed from the oral side (3), and in cross section with the mouth pointing upward (4). Expression in the oral ectoderm is consistent with a role in metamorphosis or defence against pathogenic microorganisms.

A search for genes encoding lectin domains in clusters II, III and IV identified six unigenes, two of which, A036-E7 and A049-E7, have significant overall similarity to a haemolytic lectin from sea cucumber. They lack clear *Nematostella *(or *Hydra*) counterparts, but a homologous gene is present in the Caribbean coral, *Acropora palmata *[15]. The two *A. millepora *proteins are 82.1% identical to one another (Figure 3A), and 50.4% and 48% identical to *Cucumaria echinata *CEL-III [16] respectively. These were amongst the most highly represented of the differentially expressed unigenes (A036-E7 was represented by 13 ESTs and A049-E7 by 4) and, based on their expression patterns, they are candidates for roles in metamorphosis. In situ hybridization (Figure 3B, C) revealed that both A036-E7 and A049-E7 are expressed in a subpopulation of ectodermal cells in the oral half of the larva (Figure 3B1-2; C1-2). In the post-settlement primary polyp they are exclusively expressed orally on the side that is exposed to the environment, the other, non-expressing side being against the substratum (Figure 3B3-4; C3-4). *C. echinata *CEL-III functions as an oligomer, apparently causing osmotic rupture of cell membranes after attachment to membrane-bound sugars [16,17], and their high sequence similarity suggests similar roles for the two *Acropora *proteins in cell recognition and lysis for tissue remodelling during metamorphosis. Alternatively, expression on the exposed surface of the polyp is also consistent with a role in self-defence, and could indicate a function in lysis of invading microorganisms by a similar mechanism, as suggested by Kouzuma et al [17].

### Other lectins in nematocyst differentiation

**Figure 4:**
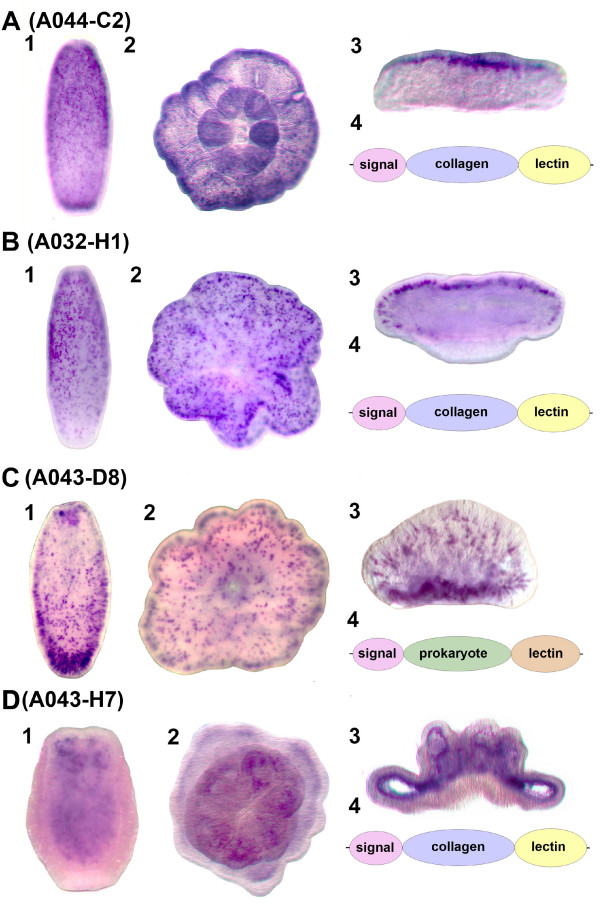
**Whole mount in situ hybridization of lectin coding genes A044-C2, A032-H1, A043-D8 and A043-H7**. Localisation of (A) A044-C2, (B) A032-H1, (C) A043-D8 and (D) A043-H7 transcripts (dark purple) in (1) presettlement planula larvae, (2) settled polyps, and (3) cross sections of the latter with the mouth pointing upward. A044-C2 and A032-H1 are expressed in vase-shaped cells abutting the mesoglea some of which are putative cnidoblasts (Additional File 3), consistent with the presence of minicollagen-like domains. A043-D8 is expressed in ectodermal cells concentrated at the aboral end of larvae, and A043-H7 is expressed endodermally, consistent with a role in symbiont uptake via the digestive system. A4-D4 are schematic representations of the protein structures of the genes (not drawn to scale). The A043-D8 protein has a type C lectin domain distinct from the galactose-binding domains in the other three proteins, hence it is represented in a different colour.

Three of the four remaining lectin domain containing proteins (A044-C2, A032-H1, and A043-H7) share an unusual structure, as they are each predicted by InterProScan [18] to contain an N-terminal signal peptide (for transport to the ER and secretion or organelle targeting), a central collagen domain, and a C-terminal galactose binding lectin domain (Figure 4). Blast searching showed that all three were most similar to *Nematostella *proteins, and structural comparisons indicate that these *Nematostella *and *Acropora *proteins, although resembling the mini-collagens known from *Hydra *[19], are thus far known only from anthozoan cnidarians. Canonical mini-collagens [19,20] are components of the walls of cnidarian nematocysts, and are defined by the presence of approximately fourteen Gly-X-Y repeats flanked by proline-rich and Cys-repeat regions. The *Acropora *molecules described here, together with *Nematostella *mini-collagen-like proteins, are distinct in also containing lectin domains; there are no *Hydra *proteins which contain both of these domains. Both A044-C2 and A032-H1 have uninterrupted minicollagen repeats, and for these, whole mount in situ hybridization revealed a common expression pattern, transcripts first appearing in scattered ectodermal cells which are more abundant toward the oral end of the planula and then becoming limited to the oral side of the post-settlement polyp (Figure 4A, B). Nematocysts are first apparent in the early planula larva (Additional File 2) and sections of embedded whole mount in situ preparations reveal expression in presumed cnidoblasts (Additional File 3), but also in other cells without the characteristic cnidoblast morphology. Whether these cells are developmental stages of cnidoblasts, or an entirely different class of cell, remains to be established. However, in the third of these related proteins, A043-H7, the minicollagen repeat is interrupted, and a completely different expression pattern is observed (see below).

Whereas the five proteins discussed above all contain galactose-binding lectin domains, the last of these six differentially expressed proteins (A043-D8) contains a C type lectin domain. Moreover, whilst a signal peptide is present, A043-D8 does not contain a mini-collagen domain. As in the case of A044-C2 and A032-H1, expression of A043-D8 appears in scattered ectodermal cells as the planula is developing (Figure 4C), although the distribution of these cells appears to differ somewhat from those shown in Figures 4A and 4B. Histological sections fail to reveal any evidence of expression in obvious cnidoblasts.

### A potential mediator of symbiont uptake

*Acropora *species acquire symbionts directly from the environment and although uptake in the wild has only been observed a few days after settlement [21], larvae of *Acropora *[22,23] and a number of other coral species [24,25] are competent to take up symbionts. However, the exact time and mode of uptake remain to be established. Lectin/polysaccharide signalling is used in many systems as a mechanism for symbiotic recognition [26], and has been implicated in the establishment of symbiosis in various marine invertebrates (e.g[27]). In the octocoral *Sinularia lochmodes *a lectin is involved in the conversion of *Symbiodinium *from a motile to the non-motile form required for symbiosis [28,29]. Also, masking cell surface glycoproteins with lectins decreases the rate of *Symbiodinium *infection of the sea anemone *Aiptasia pulchella *[30] and enzymatic digestion of cell surface glycans prevents *Symbiodinium *recognition and the establishment of symbiosis in the coral *Fungia scutaria *[31]. Although Smith [32] has argued otherwise, these more recent experiments point to a possible role for lectins in symbiont recognition/uptake in corals.

The one differentially regulated coral protein containing a lectin domain and with an expression pattern consistent with a role in symbiont uptake is A043-H7, introduced in the previous section as a mini-collagen-like protein. Unlike those in the proteins with similar domain architecture (A044-C2 and A032-H1), the mini-collagen domain of A043-H7 is interrupted, (which may have structural consequences) and the gene's expression pattern is completely different. The expression pattern of A043-H7 immediately prior to settlement (Figure 4D) is consistent with a role in symbiont uptake since, in contrast to many other cnidarians, the endoderm of the *Acropora *planula is tightly packed with yolk cells and frequently is hollow only immediately adjacent to the oral pore. As the endoderm is the most common route of cnidarian infection (see Discussion), the endodermal region immediately adjacent to the oral pore (i.e. the zone of A043-H7 expression) is a probable site of symbiont infection in the case of *Acropora *larvae. Confocal microscopy was recently used to demonstrate the binding of an *A millepora *mannose-binding lectin, which was not among our ESTs, to *Symbiodinium*, but its localization within the coral remains unknown [14].

### Conserved and novel genes with roles in calcification

The molecular basis of calcification in corals is not well understood; the process involves the deposition of calcium carbonate in an area defined by an organic matrix [33] and is initiated immediately after settlement and prior to metamorphosis [34]. Initially a flattened plate is laid down, upon which are deposited radiating vertical walls corresponding to the septa which give the polyp its six-fold symmetry. Initial calcification can, and in the case of *Acropora millepora *does, happen in the absence of *Symbiodinium*, but the massive calcification of larger colonies is dependent on the photosynthetic symbiont through interacting cycles of respiration, photosynthesis and calcification. Although many animal phyla include calcifying representatives, few components of the calcification machinery appear to be conserved between different lineages. For example, in the scleractinian *Galaxea fascicularis*, one of the most prevalent protein components of the calcifying organic matrix is galaxin [35], which appears to be unique to corals. One exception to this heterogeneity is the alpha type carbonic anhydrase family, which has been implicated in CaCO_3 _deposition from sponges to vertebrates [36]. Most animals have multiple carbonic anhydrases; distinct subfamilies are recognised [37,38] each of which is widely distributed phylogenetically, but in addition some calcifying animals have atypical carbonic anhydrases that may represent lineage specific adaptations to facilitate CaCO_3 _deposition. For example, nacrein – a soluble organic matrix protein in the nacreous layer of pearl oysters – contains a carbonic anhydrase domain that is split by a Gly-X-Asn repeat domain [39] which may have a regulatory role [40]. In a directly relevant example, Tambutte et al. [38] have recently demonstrated that active carbonic anhydrase is present in the organic matrix of *Tubastrea aurea *and plays a direct role in the calcification process. In another recent paper Moya et al [41] have cloned, sequenced and immunolocalized a previously undescribed CA from the coral *Stylophora pistillata*. It is localized in the calicoblast ectoderm, from which it is secreted, and has a CA catalytic function. In terms of understanding the bases of skeleton deposition, carbonic anhydrases are therefore of particular interest.

**Figure 5:**
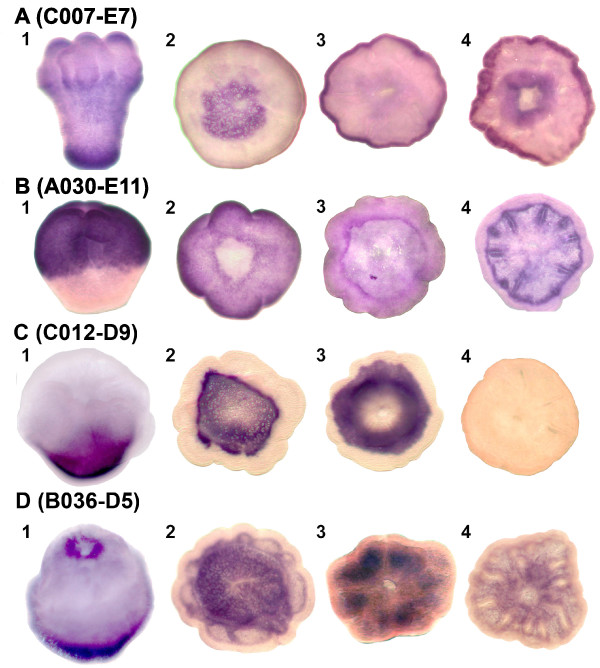
**Whole mount in situ hybridization of two carbonic anhydrases and two genes of unknown function which may be involved in calcification**. Localisation of transcripts (dark purple) of carbonic anhydrases (A) C007-E7 and (B) A030-E11 and genes of unknown function (C) C012-D9 and (D) B036-D5 are shown in (1) metamorphosing larvae and (2-4) postsettlement polyps of three different ages (2 is youngest, 4 is oldest). C007-E7, C012-D9 and B036-D5 have expression patterns consistent with involvement in early calcification as they are expressed in the aboral end of metamorphosing larvae (A1, C1, D1) and the basal disc of early postsettlement polyps (A2, C2, D2), whilst A020-E11 (B) has an expression pattern consistent with a role in later calcification, as it is expressed in the septa (B4) of the oldest polyp, where the formation of calcified adult structures occurs.

Two carbonic anhydrase genes, C007-E7 and A030-E11 (cluster III) are up-regulated in the planula larva and post-settlement stages, and in situ hybridization shows that the expression of each gene is spatially restricted at those stages of development. C007-E7 is expressed most strongly in a restricted area at the aboral end of the metamorphosing larva and primary polyp (Figure 5A1, A2). The expression of this gene in a disc at the aboral end is consistent with a role in calcification as this is the site where the process is initiated [34,42-44]. In the slightly older polyp the expression in the aboral disc decreases to a circumferential ring (Figure 5A3), and still later (Figure 5A4), this ring is maintained, and expression commences in the tentacles. This expression pattern in the basal plate is consistent with involvement of carbonic anhydrase C007-E7 in the onset of calcification, but indicates that this carbonic anhydrase is not involved in the phase of calcification during which the adult structures are formed.

The second carbonic anhydrase, A030-E11, was expressed in the oral half of the metamorphosing larva (Figure 5B1) and the entire ectoderm of the primary polyp, except the aboral disc (Figure 5B2) and the oral pore (data not shown). In older polyps this carbonic anhydrase is expressed in the septa, where calcification is occurring to form adult structures (Figure 5B4).

Expression analysis reveals that some "unique" coral genes have spatial expression patterns strikingly like that of carbonic anhydrase C007-E7, i.e. consistent with roles in the initiation of calcification. Figure 5C1 and 5D1 show genes with expression at the aboral end of the metamorphosing larva and in the basal plate of the metamorphosing larva, respectively. However, differences are apparent slightly later – C012-D9 expression becomes restricted to an aboral ring, and then appears to be switched off (Figure 5C3, C4). Whilst B036-D5 expression also appears to be down-regulated in the basal plate, transcripts can be visualised in the mesenteries (Figure 5D4) at a stage when C012-D9 transcripts are undetectable. Neither of these genes encodes known domains or could be functionally classified (using BlastP, Phi-Blast and InterPro Scan). However, their expression patterns are consistent with roles in early calcification.

### A synexpression cluster of coral-specific genes

**Figure 6:**
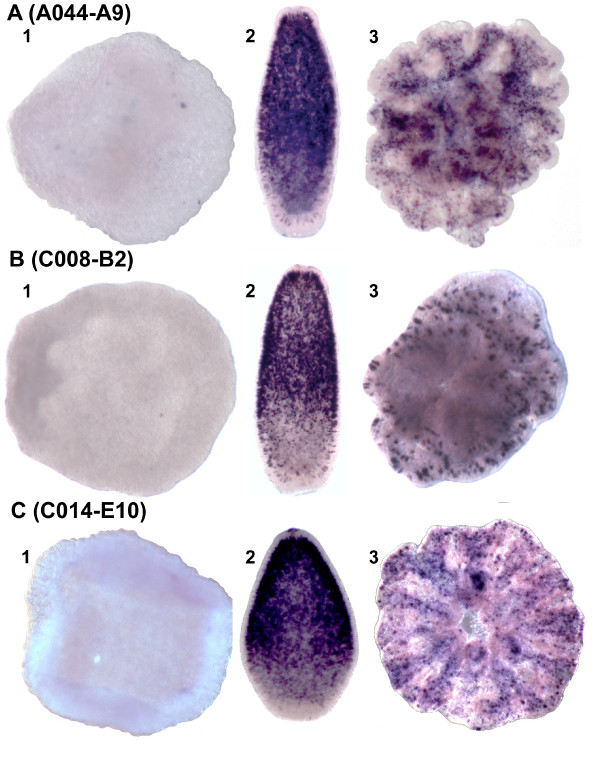
**Whole mount in situ hybridization of three genes of unknown function**. In addition to the temporal synexpression established by microarray these three genes share common expression patterns and thus form a temporo-spatial synexpression group. Localisation of (A) A044-A9, (B) C008-B2 and (C) C014-E10 transcripts (dark purple) in (1) prawnchip, (2) presettlement larva, and (3) postsettlement polyp. Orientation in presettlement and postsettlement larvae is oral upward. Lack of expression in the prawnchip is followed by expression in a subset of ectodermal cells concentrated at the oral end of the presettlement larvae and postsettlement polyps. Their synexpression both temporally and spatially, suggest that they may be a novel group of genes interacting with one another.

As indicated above, the proportion of unique genes was highest in synexpression clusters II ('planula') and IV ('primary polyp'). To investigate their possible roles, in situ expression patterns were determined for many of these coral-specific genes. Many gave specific expression patterns, some of which are consistent with roles in processes such as calcification, as previously discussed. In other cases, although groups of "unknown" genes appear to be expressed in the same cells, it is more difficult to interpret the likely biological significance of the patterns. One example of this phenomenon is provided by three 'planula' cluster unigenes (A044-A9, C008-B2 and C014-E10) with no clear hits to genes in other organisms; the corresponding proteins are each predicted to contain a signal peptide, and C014-E10 contains a SEA domain (an extracellular domain involved in carbohydrate binding). In situ analysis showed that in the planula, the three transcripts are co-localised in a subpopulation of ectodermal cells that is concentrated orally. The post-settlement expression patterns of these three genes were also very similar, transcripts in each case being localised in scattered ectodermal cells of the polyp (Figure 6A–C). The apparent co-localisation and co-expression of these unrelated but unique unigenes suggests that they may function in a common process or signaling pathway. The size of the synexpression group to which these three genes belong is unknown, but such gene clusters are of great interest, since they may represent coral-specific pathways or functions. Unfortunately, such genes also present great analytical difficulties; since their lack of clear homologs limits the inference of function from structure and the molecular tools required to test function are not yet available in corals, although progress is being made in that direction with other cnidarians [45-47].

## Discussion and conclusion

### Validation of the approach and methodology

Virtual northern blots for eight genes were consistent with the microarray results, thus confirming them. In addition, and consistent with the microarray results being accurate, several mini-collagen-like proteins were upregulated in the planula. Mini-collagens have thus far only been described from nematocysts, cnidarian-specific structures which first appear at the planula stage in *A. millepora *(Additional Files 2, 3).

### Taxonomic and functional breakdown of the genes

The composition of the EST set used in these microarray experiments has previously been considered specifically with respect to the complement of developmental signalling pathway components [2,3], but this paper is the first to examine broad scale changes in gene expression during development for any cnidarian. The use of different criteria and thresholds, and the ever-changing baseline provided by the databases, complicates making direct comparisons with other developmental studies. For example, although a recent paper on developmental gene expression in the ascidian, *Molgula *[48] addressed many of the same questions, it focussed specifically on highly expressed genes (i.e. only those accounting for more than 0.2% of the total number of ESTs) so it is not possible to interpret apparent differences, such as in the percentage of unique genes. In terms of developmental changes, it is particularly noteworthy that the percentage of "core" genes (59%; i.e. those genes shared with members of other kingdoms as well as other animals) is highest in cluster VI and that the percentage of unique genes (12%) is lowest in cluster I. Presumably these figures reflect shifts from common cellular pathways during very early development to greater cellular and molecular diversification later. As in many other animals, the early development of *Acropora *appears to involve many stored maternal mRNAs. The composition of the maternal mRNA pool is complex, consisting principally of low abundance transcripts including those involved with cell division, RNA metabolism, and regulation of gene transcription (L McFarlane, unpublished). Among genes of particular interest, H2A.Z and H1, histones with roles in priming chromatin for developmental gene expression [49] in a variety of other systems, are highly represented in the prawn chip ESTs and strongly down regulated thereafter, as are cyclins A and B3. In *Drosophila *and *Xenopus*, maternal cyclin transcript levels are initially very high and then decrease dramatically after the onset of gastrulation [50-52]. *Acropora *may therefore follow this pattern of abundant maternal cyclin transcripts that drive very rapid cell proliferation early in embryogenesis, followed by lower transcript levels with the onset of slower developmentally regulated cell cycles. Cell cycle transcripts such as cyclin A and B were also abundant among the cleaving embryo ESTs of *Molgula tectiformis *[48] and in pre-gastrulation stages of *Xenopus *[53] and *Drosophila *[54].

### Lectin domain proteins are potentially involved in diverse processes

There are a number of precedents for the involvement of lectin-containing proteins in metamorphosis. Lectins are differentially expressed at metamorphosis in two ascidians, *Herdmania curvata *[55] and *Boltenia villosa *[11,12]. In *Boltenia*, four lectins and two key lectin pathway genes are up-regulated in the larva or the newly settled adult [11]. The lectin induced complement pathway, which is initiated by a mannose-binding lectin, is important in *Boltenia *for the recognition of those bacteria which induce metamorphosis and tissue remodeling [12]. It is possible that the lectins up-regulated at metamorphosis in *Acropora *have an analogous role in activating tissue remodelling. Consistent with this idea, a possible complement effector, the perforin domain protein apextrin, is expressed in a strikingly similar pattern to those of the CELIII lectins during metamorphosis in *Acropora *[56].

Lectin domain-containing proteins also potentially function in the recognition of symbionts by corals. Lectin/polysaccharide signalling is used in many systems as a mechanism for symbiont recognition, the most widely known example being the recognition of sugars on the surface of nitrogen-fixing bacteria by the lectins of their host legume during the establishment of their symbiosis. *Symbiodinium *in scleractinian corals reside in the endoderm, and two mechanisms of entry have been described in those corals that acquire them from the environment. The first is directly into the endoderm via the oral pore after it is formed 3–5 days post fertilization in association with feeding, as was demonstrated in the coral *Fungia scutaria *[25] and the anemone *Anthopleura elegantissima *[57]. The second, also demonstrated in *Fungia *[24], is that they can enter via the epithelium pre- or post-gastrulation. Those which have entered by the ectoderm are then shunted to the endoderm where they are retained [24]. Elegant studies in the latter half of the last century described the cell biology of symbiont uptake and retention, for example [58], and it has recently been established that members of the Rab family of proteins are involved in determining whether symbionts are digested or retained [59-61]. *Symbiodinium *are not transmitted through the eggs of *A. millepora*, and while planulae can be infected [23] this may only occur after the oral pore has opened shortly before settlement ([22] and AH Baird, pers comm.) although the timing and mode of symbiont uptake remain to be firmly established. The limited available field observations indicate that infection normally does not occur until a few days after settlement in *A. millepora *[21]. These observations point to the endoderm as the likeliest point of *Symbiodinium *uptake, but do not rule out a possible role for the ectoderm. There is clear evidence from a number of cnidarian species of selective maintenance of the most "appropriate" clade of symbiont, while conclusions on specificity of uptake and its possible mechanisms are equivocal, perhaps due to interspecific variabity. Nevertheless, there is evidence that lectins function in symbiont recognition, as previously summarised, and these molecules therefore remain obvious candidates for roles in symbiont uptake and maintenance by *Acropora*.

### Genes involved in calcification

Two alpha type carbonic anhydrases are expressed in patterns that are consistent with roles in calcification. However, these genes are not restricted to heavily calcifying cnidarians, as both have probable orthologs in sea anemones and other cnidarians. This is perhaps not surprising, as carbonic anhydrases are involved in pH and CO_2_/bicarbonate homeostasis in all organisms, and the ability to deposit some form of calcified exoskeleton is taxonomically widespread among cnidarians. For example, polyps of the hydrozoan *Hydractinia symbiolongicarpus *secrete a mat of calcium carbonate, in the form of aragonite, on their substrate [62]. Two membrane-associated carbonic anhydrases have been described from planulae of the coral *Fungia scutaria*, but they are short and missing amino acids thought to be necessary for CA activity, although the authors hypothesize that they could play a role in the onset of calcification at the time of settlement [63]. The first *Acropora *carbonic anhydrase, C007-E7, matches most strongly to vertebrate IV/XV-type carbonic anhydrases, and consistent with this, is predicted to be GPI anchored. C007-E7 has likely orthologs in both *Nematostella *and *Hydra*. The second carbonic anhydrase, A30-E11, is a I/II-type carbonic anhydrase and is likely to be the *Acropora *ortholog of a protein identified in the sea anemone, *Anthopleura elegantissima *(29.8% identity and 43.1% similarity) as a "symbiosis gene" – it is strongly up-regulated when this facultatively symbiotic anemone takes up endosymbionts [64]. However, clear counterparts of this soluble cytosolic type carbonic anhydrase are present in both *Nematostella *and *Hydra magnipapillata*, neither of which harbours symbionts. Whereas the two carbonic anhydrase genes are not restricted to calcifying cnidarians, a number of other coral genes with similar expression patterns have no apparent sea anemone or *Hydra *homologs. One possible scenario is that many of the genes involved in calcium processing will have a widespread distribution while some of those involved in secreting the organic matrix may be more specific, as in the case of galaxin. It will be particularly interesting to see whether different gene repertoires play a significant part in the determining the dramatic differences in colony morphology that are characteristic of the various corals or whether this is due mainly to deploying the same genes in different ways.

### "Coral-specific" processes as variations on known themes

One conclusion that follows from the work presented above is that many of the molecules involved in "coral-specific" processes such as metamorphosis and calcification are not coral specific – genes whose expression patterns imply key roles in implementing metamorphosis, such as the lectins A036-E7 and A049-E7 and apextrin [56] have homologs in other animals even though they are not present in *Nematostella*. Both of the carbonic anhydrases implicated in calcification also have clear counterparts in non-calcifying cnidarians. A second conclusion is that processes central to coral biology, such as symbiont recognition, may have analogous biochemical bases in phylogenetically distant systems. Lectins function in symbiont recognition in the legume-*Rhizobium *system; this analogy may be useful in understanding how specificity might be achieved in the coral/dinoflagellate symbiosis and in exploring the roles of the candidate molecules identified here. As in ascidians, metamorphosis in *Acropora *involves activation of an innate immune response, as both lectins and the perforin domain protein apextrin are strongly and specifically expressed at this time. Inevitably, other genes implicated in coral-specific processes appear at this stage to be taxon-restricted, but it is unclear to what extent this simply reflects the limited number and range of animals for which whole genome data are yet available. Genes that are today considered "coral-specific" may actually be more widely distributed; the number of genes considered vertebrate-specific shrinks with the publication of each additional animal whole genome sequence. Moreover, genes with no clear homologs may simply be old genes that have evolved beyond recognition.

One promising approach arises from the prediction that genes involved in "coral-specific" processes such as symbiont recognition are under positive selection. With the imminent availability of large EST datasets for several corals, a combination of in silico and in situ approaches should identify these genes and build on the pioneering study reported here.

## Methods

### Microarray description

The microarrays used in this experiment consisted of 13,392 spots derived from 12,240 cDNA clones (1,152 clones are represented more than once) and 432 spots representing positive and negative controls. The cDNA clones spotted onto the array were randomly selected from cDNA libraries that had been constructed in Lambda ZAP (Stratagene), and include 3456 clones from the prawnchip developmental stage, 4608 clones from the planula larva stage [65], and 4128 clones from the primary polyp. All of the material used for making the libraries came from Nelly Bay, Magnetic Island, Queensland, Australia (19°08'S 146°50'E).

All cDNAs spotted onto the slides were derived from cDNA libraries of the appropriate developmental stages. They were isolated by TempliPhi (GE Life Sciences) on excised clones except for 2,000 postsettlement polyp clones which were PCR amplified directly from individual phage suspensions and 3,012 planula larva cDNAs which were isolated previously [2]

### Generation and spotting of cDNAs

PCR (1× HotMaster Taq Reaction buffer, 0.25 mM each dNTP, 25 pmoles of each of M13 Forward and M13 Reverse primer, 1.25 units of HotMaster Taq Polymerase (Eppendorf) spiked with Pfu (Promega) in a 25 ul reaction) was used to generate DNA for spotting onto microarray slides. Phage suspension was used as template by adding 4 ul to the PCR mix. TempliPhi was used as a template by dipping a pin into the TempliPhi reaction and then into the PCR mix. PCR was carried out in 96-well plates (ABGene) under the following conditions: 94°C for 30 s, 50°C for 30 s and 72°C for 1 min, for 30–35 cycles. PCR products were purified using 96 well Multiscreen plates (Millipore).

Microarrays were generated by spotting the amplified cDNA onto GAPSII slides using a Biorad Chipwriter Pro, and then fixed by UV light exposure (150 mJ) followed by baking at 80°C for 3 hours. All cDNA clones represented on the arrays were sequenced from the 5' direction using standard Sanger (ABI Big Dye) sequencing technology.

### EST analyses

After data filtering, ESTs were clustered using CAP3 [66]. The coding potential of the resulting unigenes was analysed using ESTScan [67]. 5081 were predicted to give rise to bonafide proteins, using the criterion of a coding potential of 25 or greater. The EST contigs which had predicted peptides were used to search the Uniprot database using BlastX [68] with a threshold of e = 1 × 10^-5 ^in order to functionally classify the predicted proteins according to the scheme in [10].

### Experimental design

**Figure 7:**
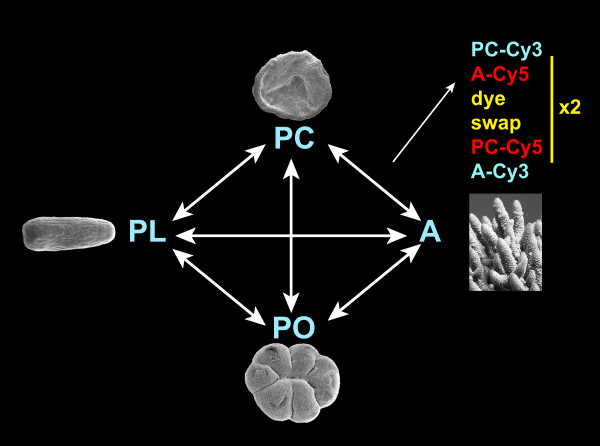
**Microarray experimental design**. Each developmental stage used in this experiment was directly compared to all others. Each arrow represents four hybridizations; two in one dye orientation (Cy3–Cy5) and two in the other (Cy5–Cy3), hence 24 slides were used in total. Further details are given in Materials and Methods.

To assay for changes in gene expression during *Acropora *development, mRNA was isolated from four different developmental stages: the pre-gastrula "prawn chip" stage (8 hpf), the planula larva stage (83 hpf), the post-settlement primary polyp (130 hpf) and the adult colony. The rationale for selecting these stages is that they span key developmental events including the establishment of tissue layers and body axes at gastrulation, the transduction of settlement cues, settlement and metamorphosis, and the initiation of calcification and uptake of symbionts. Prawn chips, planula larvae and primary polyps were the offspring of colonies collected from Nelly Bay, Magnetic Island (19°08'S 146°50'E). Adult tissue was obtained from a colony in the same bay. Pools of approximately 1000 embryos were made to create each biological replicate [69]. Total RNA was extracted from these for each of our stage specific 'targets'. Tissue from a single colony was used in the case of adult RNA extraction. The entire experiment was replicated on different days using separate collections of material thus giving two biological replicates. Within each biological replicate, each developmental stage was compared with every other twice; once in each dye orientation. Thus, there are two biological and two technical replicates for each comparison (Figure 7). Since there are six possible comparisons with this design the entire experiment used 24 slides – 12 for each biological replicate.

cDNA for probing arrays was produced from unamplified total RNA which was extracted using TRI Reagent (Ambion) according to the manufacturer's instructions. The quality was assessed using denaturing gel electrophoresis using standard methods [70]. For each hybridized sample, total RNA (80 ug) was reverse transcribed, labelled and hybridised using standard protocols [71].

### Data analysis and verification

Slides were scanned using a GenePix 4200A scanner, and data extracted using Spot [72]. All further analyses were carried out using the limma package [73] for the R system [74]. Print-tip loess normalisation [75] was performed on each slide. Quantile normalisation was applied to mean log-intensities in order to make the distributions essentially the same across arrays.

The methodology used for statistical analysis is described in Smyth [76]. The prior probability of differential expression, for each pair of comparisons between stages, was taken as 0.1. The Benjamini and Hochberg method [77] was used to adjust the sequence-wise p-values, so that a choice of sequences for which the adjusted p-value is at most 0.05 identifies a set of differentially expressed genes in which 5% may be falsely identified as differentially expressed (see Additional File 4 for more detail). Array data have been deposited in the Gene Expression Omnibus (GEO) database (accession number GSE11251).

Results were also verified using M vs A plots, where M = the log ratio of the spot fluorescence intensity values and A = the log of the average spot fluorescence intensity. An example is given in Additional File 5. Spots for which no fluorescence was expected, including salmon sperm DNA, empty vector and primers, plotted near the origin of the MA plot, as expected. Negative controls for differential expression (i.e. spots expected to show hybridization but no differential expression), had an M value of or near to zero, but ranged in fluorescence intensity, also in accordance with expectations. Differentially expressed positive controls (i.e, spots expected to show both hybridization and differential expression between presettlement and postsettlement on the basis of virtual northern results) were positioned on either side of an M value of zero with a range of fluorescence intensities.

Cluster analysis was used to search for clusters of expression profiles in the data. K-means clustering was used to split the genes into 6 groups of differential expression profiles. Clustering was carried out using Cluster 3.0 [78] and the results viewed with Java TreeView [79]. Unigenes with protein coding potential > 25 and p-value < 0.05 in the test for differential expression between temporally sequential developmental stages were removed prior to cluster analysis.

Results for the microarray experiments were verified using "virtual northern blots" which were made using the Clontech SMART cDNA Synthesis Kit, according to the manufacturer's instructions using RNA from the same stages used in the microarray experiment. DNA used to probe the blots was generated by PCR (see section 2.5.4 PCR and spotting of cDNAs), purified using the Qiagen PCR Purification kit according to the manufacturer's instructions, and radiolabelled with ^32^P-dATP using the Prime-A-Gene Labeling System (Promega) according to the manufacturer's instructions. Hybridization was conducted according to standard protocols [70] and visualized by exposure to a Phosphorimager (Molecular Dynamics) cassette overnight. Digital images were viewed with Quantity One software.

### Low-throughput sequencing

In order to obtain the entire open reading frame, some unigenes selected for in situ hybridization required further sequencing. This was done either as described for EST sequencing or using 300 ng of plasmid as template. Raw data were viewed and edited with Chromas Lite and sequences were aligned with LaserGene (DNASTAR). cDNA sequences for genes characterized by in situ hybridization have been deposited in GenBank under accession numbers EU863776–EU863788.

### In situ hybridization

Templates for riboprobe production were generated by PCR. Riboprobe synthesis and in situ hybridization were performed as reported by [80]. In order to view further histological detail embryos stained in whole mount were embedded in LR White Resin sectioned at various thicknesses and counterstained with Saffranin O.

## Authors' contributions

LG played a major role in all aspects of the work, JM contributed to experimental design and carried out the data analysis, DH made the libraries and contributed to all other aspects of the work, SR did the large scale data comparisons, RS contributed to experimental design and writing of the manuscript, DM advised on all aspects of the work with major contributions on coral biology and writing, EB coordinated the project, with major contributions to SEM, anatomy and writing.

## Supplementary Material

Additional file 1 **Virtual northern blots for verification of microarray data**. Virtual northern blots (upper) paired with corrresponding graphical representations of microarray data for the same gene (below) with fold change in intensity of expression indicated on the Y axis. Numbers on virtual northern lanes correspond to the following stages: 1 = egg, 2 = PC, 3 = PL, 4 = PO, 5 = A. Lanes with no number are stages intermediate to those used in the microarray analysis. Genes used for virtual northern blots were chosen arbitrarily purely for verification purposes. The dotted line equates to 1.8 kb Click here for file

Additional file 2 **Stained sections of *Acropora millepora *embryos reveal the stage at which nematocysts develop**. Nematocysts first appear between the prawn chip (PC) and planula (PL) stages. (A) Transverse section of a PC, showing uniform cells with little overt sign of differentiation. (B) Trichrome-stained planula larva showing many differentiated cell types in the ectoderm. (C) Enlargement of planula ectoderm, showing a differentiated spirocyst or nematocyst (N). Click here for file

Additional file 3 **Sectioned embryos following in situ hybridization with A032-H1 and Safranin O staining**. Stained sections of embryos embedded following whole mount in-situ hybridization and counterstaining with Safranin O, show labelled cnidoblasts near the base of the the ectoderm. (A) low magnification micrograph for orientation, (B-D) through focus series through a similar cell showing that the message appears to be restricted to the periphery of the cell. Click here for file

Additional file 4 **Details of microarray data analysis**. This file contains additional detail about the microarray data analysis and includes the limma code used in the analysis. Click here for file

Additional file 5 **MA plot for one slide**. This graph plots M (= log2(ratio of intensities)) vs A (= log2(product of intensities)) and is a diagnostic plot to check for successful normalization. The data on this slide have been normalized successfully as the data points are centred around an M value of zero, that is, there is no dye bias. cDNAs are represented by black spots, whilst negative controls are blue, and positive controls are red. Click here for file
