# Supplementary figures and images for: Microarray analysis identifies candidate genes for key roles in coral development
Source: BMC Genomics. 2008 Nov 14;9:540. doi: 10.1186/1471-2164-9-540 (PMC2629781; doi:10.1186/1471-2164-9-540)

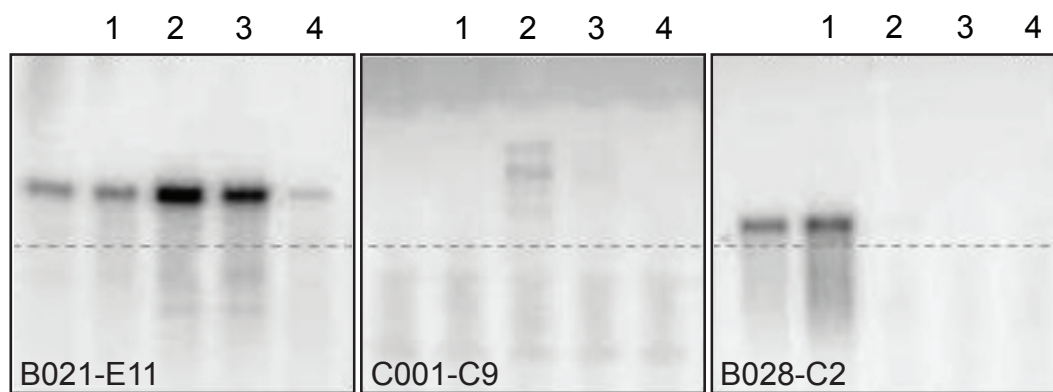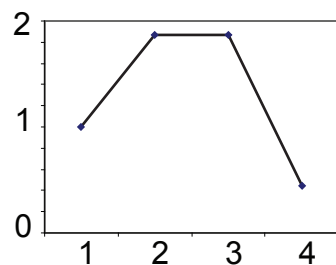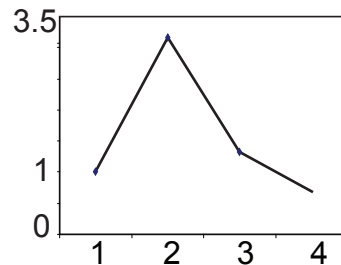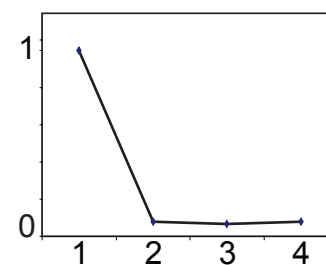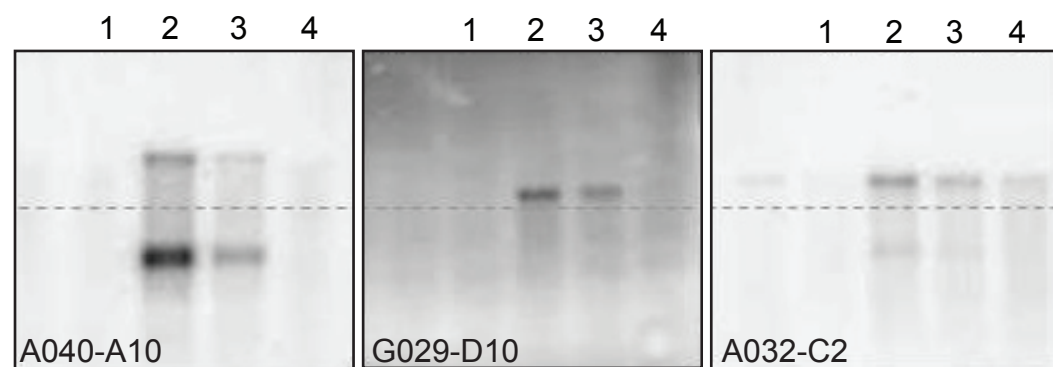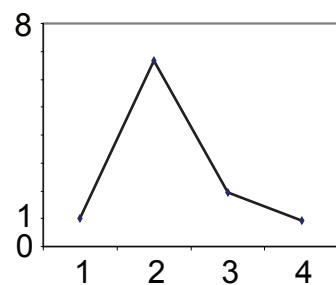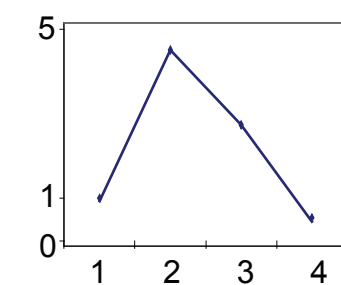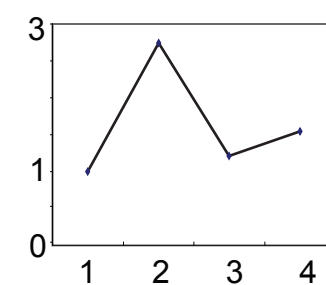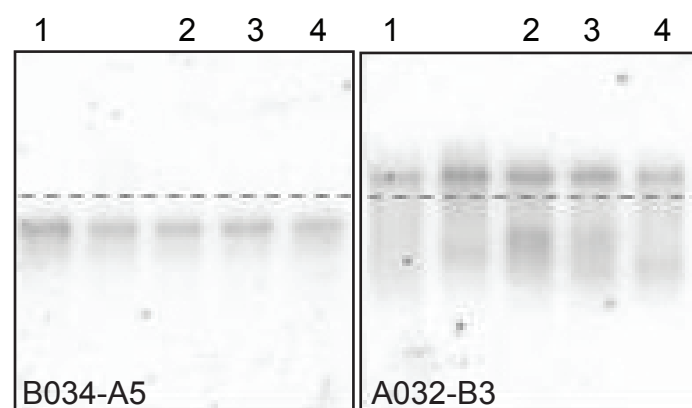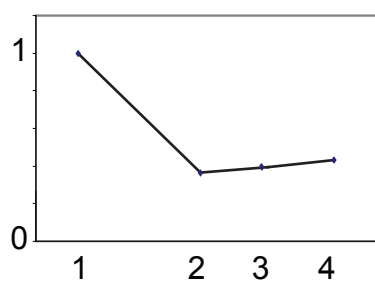

Not Differentially expressed

Supplement: Additional file 1 — Virtual northern blots for verification of microarray data. Virtual northern blots (upper) paired with corrresponding graphical representations of microarray data for the same gene (below) with fold change in intensity of expression indicated on the Y axis. Numbers on virtual northern lanes correspond to the following stages: 1 = egg, 2 = PC, 3 = PL, 4 = PO, 5 = A. Lanes with no number are stages intermediate to those used in the microarray analysis. Genes used for virtual northern blots were chosen arbitrarily purely for verification purposes. The dotted line equates to 1.8 kb [file 1471-2164-9-540-S1.pdf]

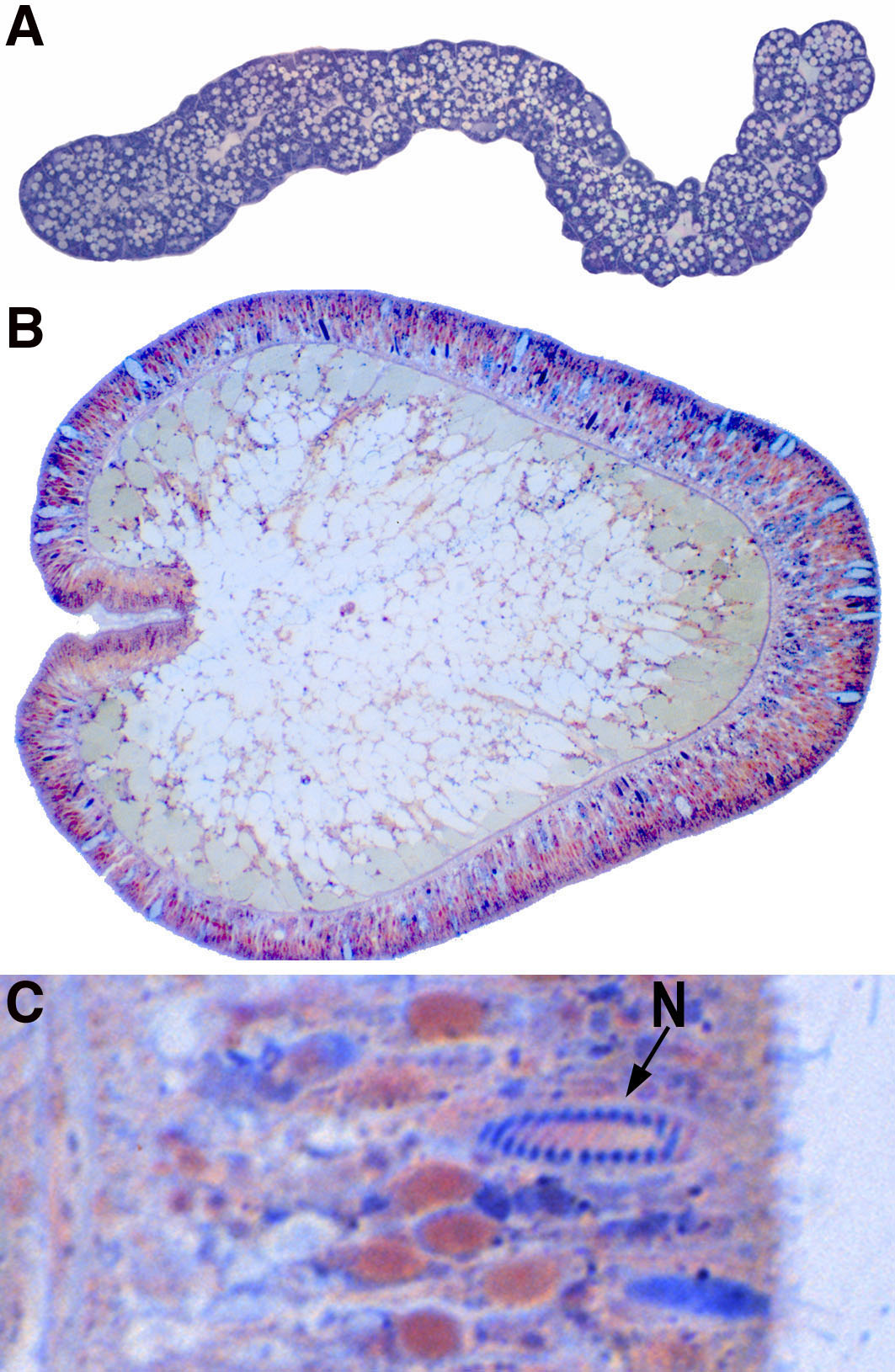

Supplement: Additional file 2 — Stained sections of Acropora millepora embryos reveal the stage at which nematocysts develop. Nematocysts first appear between the prawn chip (PC) and planula (PL) stages. (A) Transverse section of a PC, showing uniform cells with little overt sign of differentiation. (B) Trichrome-stained planula larva showing many differentiated cell types in the ectoderm. (C) Enlargement of planula ectoderm, showing a differentiated spirocyst or nematocyst (N). [file 1471-2164-9-540-S2.jpeg]

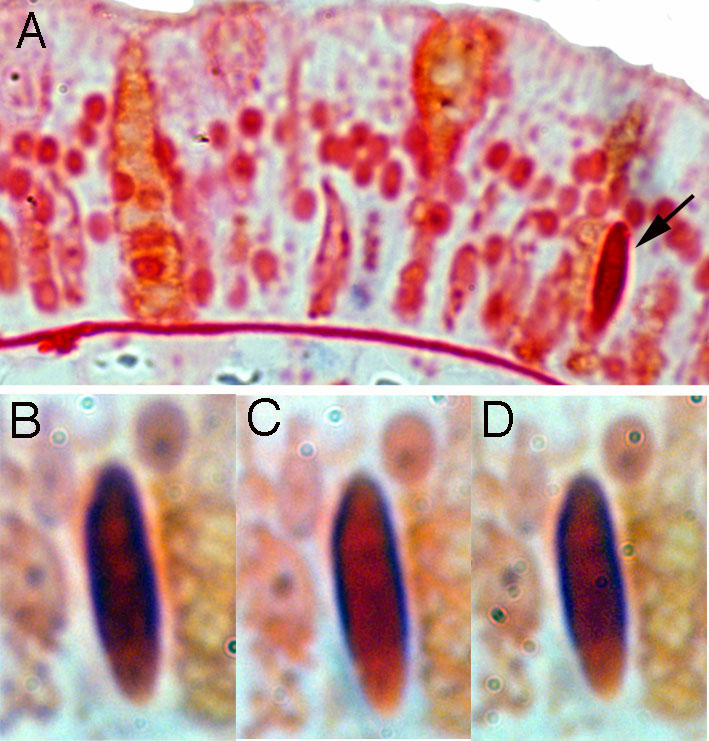

Supplement: Additional file 3 — Sectioned embryos following in situ hybridization with A032-H1 and Safranin O staining. Stained sections of embryos embedded following whole mount in-situ hybridization and counterstaining with Safranin O, show labelled cnidoblasts near the base of the the ectoderm. (A) low magnification micrograph for orientation, (B-D) through focus series through a similar cell showing that the message appears to be restricted to the periphery of the cell. [file 1471-2164-9-540-S3.jpeg]

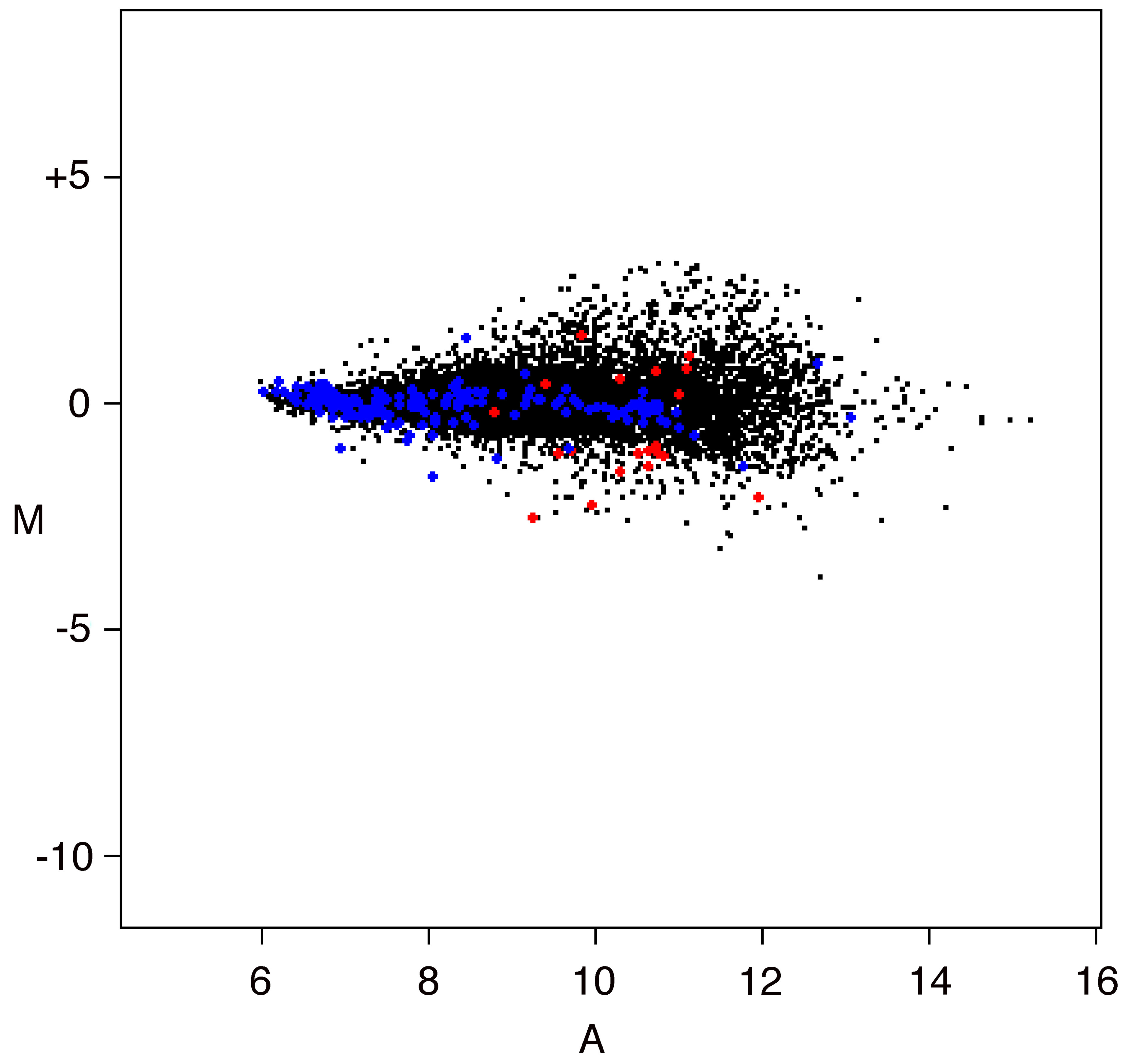

Supplement: Additional file 5 — MA plot for one slide. This graph plots M (= log2(ratio of intensities)) vs A (= log2(product of intensities)) and is a diagnostic plot to check for successful normalization. The data on this slide have been normalized successfully as the data points are centred around an M value of zero, that is, there is no dye bias. cDNAs are represented by black spots, whilst negative controls are blue, and positive controls are red. [file 1471-2164-9-540-S5.jpeg]
