# Supplementary material for: Microarray analysis identifies candidate genes for key roles in coral development
Source: BMC Genomics. 2008 Nov 14;9:540. doi: 10.1186/1471-2164-9-540 (PMC2629781; doi:10.1186/1471-2164-9-540)
Supplement: Additional file 4 — Details of microarray data analysis. This file contains additional detail about the microarray data analysis and includes the limma code used in the analysis. [file 1471-2164-9-540-S4.doc]

**S4 Details of microarray data analysis**

It was assumed that the four hybridizations sampled from the same source of statistical variation have a redundancy that allows us to estimate an error variance, since for each developmental stage, there was a direct estimate of C-A, and an indirect estimate as (C-B)+(B-A). The R code used for the analysis is as below:

library(limma)

targets <- readTargets("sTargets.txt")

targets$FileName

targets

coralSRG <- read.maimages(targets$FileName, source = "spot", other.columns=list(area="area", badspot="badspot"))

coralSRG$genes <- readGAL()

coralSRG$printer <- getLayout(coralSRG$genes)

coralSRG$printer

spottypes<-readSpotTypes()

coralSRG$genes$Status <- controlStatus(spottypes, coralSRG)

param <- matrix(c(One2=c(-1,1,0,0),Two3=c(0,-1,1,0),Three4=c(0,0,-1,1)), ncol=3)

rownames(param) <- uniqueTargets(targets)[c(3,4,2,1)]

colnames(param) <- c("One2", "Two3", "Three4")

design <- modelMatrix(targets, parameters=param)

contrast.matrix <- cbind(one2=c(1,0,0), two3=c(0,1,0), three4=c(0,0,1), one3=c(1,1,0), one4=c(1,1,1), two4=c(0,1,1))

rawMAcoralSRG <- normalizeWithinArrays(coralSRG, method = "none")

plotMA3by2(rawMAcoralSRG)

MAcoralSRG <- normalizeWithinArrays(coralSRG, method = "printtiploess")

plotMA3by2(MAcoralSRG)

boxplot(MAcoralSRG$M ~ col(MAcoralSRG$M), names = colnames(MAcoralSRG$M))

nMAcoralSRG <- normalizeBetweenArrays(MAcoralSRG)

boxplot(nMAcoralSRG$M ~ col(nMAcoralSRG$M), names = colnames(nMAcoralSRG$M))

wanted <- coralSRG$genes$Status == "diff-exp ctl"

deM <- nMA$M[wanted, ]

pairs(deM)

odesign <- design

omit.slides <- c(2, 3, 12+c(2))

odesign[omit.slides,] <- 0col

ofit <- lmFit(nMAcoralSRG,odesign)

cofit <- contrasts.fit(ofit,contrast.matrix)

efit.alls <- eBayes(cofit, proportion=0.05)

sink("stage2vsstage1coralSRG.txt")

topTable(efit.alls, coef=1, number=13824)

sink()

sink("stage3vsstage2coralSRG.txt")

topTable(efit.alls, coef=2, number=13824)

sink()

sink("stage4vsstage3coralSRG.txt")

topTable(efit.alls, coef=3, number=13824)

sink()

Where the targets file was:

SlideNumber FileName Cy3 Cy5

Am002207 hybone001s.spot post prawn

Am002208 hybone002s.spot prawn adult

Am002209 hybone003s.spot pre post

Am002210 hybone004s.spot prawn post

Am002211 hybone005s.spot pre adult

Am002212 hybone006s.spot adult pre

Am002213 hybone007s.spot adult prawn

Am002214 hybone008s.spot post adult

Am002215 hybone009s.spot pre prawn

Am002216 hybone010s.spot post pre

Am002217 hybone011s.spot adult post

Am002218 hybone012s.spot prawn pre

Am002219 hybtwo001s.spot pre post

Am002200 hybtwo002s.spot adult prawn

Am002221 hybtwo003s.spot prawn pre

Am002222 hybtwo004s.spot post pre

Am002223 hybtwo005s.spot prawn post

Am002224 hybtwo006s.spot post adult

Am002225 hybtwo007s.spot adult post

Am002226 hybtwo008s.spot pre prawn

Am002227 hybtwo009s.spot adult pre

Am002228 hybtwo010s.spot prawn adult

Am002229 hybtwo011s.spot post prawn

Am002230 hybtwo012s.spot pre adult

And the spot types files was:

SpotType ID Name Colour

gene * * black

diff-exp ctl DH* * blue

96 pooled PCR Mpool * brown

96 pooled PCR M pool * brown

salmon sperm ssDNA * yellow

DA(pin ctl) DA* * brown

DB(block ctl) DB* * brown

polyA DNA PA * yellow

coral gDNA G * brown

human gDNA Cot-1 * yellow

yeast tRNA YtRNA * yellow

empty vector V * yellow

Fwd primer PFwd * yellow

Rev primer PRev * yellow
